# A Case of Placenta Prævia Treated by the Tampon and Version

**Published:** 1886-03-20

**Authors:** Henry C. Archibald

**Affiliations:** Philadelphia


### A CASE OF PLACENTA PRÆVIA TREATED BY THE TAMPON AND VERSION.

BY HENRY C. ARCHIBALD, M. D., OF PHILADELPHIA.

Upon referring to my case-book I find on the 20th of September I was called in haste to attend a Mrs. S. in what was supposed to be an attempt at miscarriage. She informed me that she was the mother of three children, all living, and never before had a miscarriage, or even an attempt at one, but that for two or three weeks past she had noticed slight evidence of blood oozing out of the vagina, and very naturally attributed it to excessive labor superinduced by moving, she having a few weeks before lost her husband from phthisis, and the worry incident to his death, together with the care of a large family without proper means for their maintenance, was the cause she assigned for what she and her immediate friends thought was a miscarriage. Upon examining, digitally, the womb, I found the os patulous and dilated about the size of a silver quarter, and thought I detected the placenta. Blood was oozing from the womb. I simply ordered one-fourth grain doses of morphia three times a day and rest in the recumbent position. Upon my next visit, on the following day, I found the bleeding had ceased and more careful examination convinced me that I had a true case of placenta prævia to deal with. Being positive in my diagnosis, I informed the nurse of the nature of the case, together with its attendant dangers, and simply advised, upon the slightest evidence of bleeding, to place her patient in bed, and give the morphia as previously directed, and that if a case uncontrollable hemorrhage should set in at any time to at once send for me; and further, that Mrs. S. would, in all probability, carry the child to full term, but have repeated attempts at hemorrhage. With these directions, I left the case. although hearing often that my advice had to be resorted to quite frequently (or, as the nurse put it, if it was not for that medicine she would flow to death).

On the 20th of November I was again hurriedly called to see Mrs. S., and found her lying in a pool of blood, head low, hips elevated. Making a hurried examination, and being satisfied that the membranes were intact, I proceeded to at once tampon, which I did with a weak solution of sub-sulphate of iron and absorbent cotton, and again gave my patient morphia to quiet the pains she was having, and then sent for my valued friend Dr. H. D. Benner, residing at Third and Christian streets, in order to obtain his valued aid and advice in the further management of this case. And although he was aware that he would receive no remuneration for any service he would render, he gladly and promptly responded to my call, and, as the sequel will show, rendered such valued aid, that he will ever have my lasting gratitude. Upon Dr. B.'s arrival, I told him the history of the case throughout, and by his advice I remained passive and awaited developments, he having approved of what I had already done (if I was certain as to its being placenta prævia, and the membranes intact).

I watched the case faithfully for three days, changing the tampon every twenty-four hours, although fearful each time I removed it, as the hemorrhage was excessive. On the third day I was again hurriedly summoned at 3 a. m., and found that labor had set in. So again sent for Dr. Benner, who promptly came again to my call. I at once removed the tampons, and for the first time Dr. Benner made an examination. He found, as I had previously, the placenta fully covering the os—the os dilatable. Version was at once suggested and performed by Dr. B., who removed the placenta from its attachment sufficient to reach the membranes, ruptured them, and seizing the child by the feet, delivered a fine healthy boy. The womb contracted as the fœtus was drawn out, and the placenta removed by pressure on the fundus. Both mother and child are doing well.

The point I desire to make in this communication is this, that in placenta prævia, when you are satisfied that the membranes are intact, the proper course is to tampon tightly, wait for labor to set in, and if the head presents, separate the placenta, rupture the membranes, and perform version.—*Medical & Surgical Reporter.*

